# Role of TRPV1 in High Temperature-Induced Mitochondrial Biogenesis in Skeletal Muscle: A Mini Review

**DOI:** 10.3389/fcell.2022.882578

**Published:** 2022-04-05

**Authors:** Yixiao Xu, Yongcai Zhao, Binghong Gao

**Affiliations:** ^1^ School of Kinesiology, Shanghai University of Sport, Shanghai, China; ^2^ College of Social Sport and Health Sciences, Tianjin University of Sport, Tianjin, China; ^3^ School of Physical Education and Training, Shanghai University of Sport, Shanghai, China

**Keywords:** transient receptor potential vanilloid 1 (TRPV1), high temperature, Ca^2+^, mitochondrial biogenesis, skeletal muscle

## Abstract

Transient receptor potential vanilloid 1 (TRPV1) is a protein that is susceptible to cell environment temperature. High temperatures of 40–45°C can activate the TRPV1 channel. TRPV1 is highly expressed in skeletal muscle and located on the sarcoplasmic reticulum (SR). Therefore, TRPV1 activated by high-temperature stress releases Ca^2+^ from the SR to the cytoplasm. Cellular Ca^2+^ accumulation is a key event that enhances TRPV1 activity by directly binding to the N-terminus and C-terminus. Moreover, Ca^2+^ is the key messenger involved in regulating mitochondrial biogenesis in skeletal muscle. Long-term activation of TRPV1 may promote mitochondrial biogenesis in skeletal muscle through the Ca^2+^-CaMKII-p38 MAPK-PGC-1α signaling axis. The discovery of the TRPV1 channel highlights the potential mechanism for high-temperature stress improving muscle mitochondrial biogenesis. The appropriate hot stimulus in thermal environments might be beneficial to the muscular mitochondrial adaptation for aerobic capacity. However, the investigation of TRPV1 on mitochondrial biogenesis is at an early stage. Further investigations need to examine the role of TRPV1 in response to mitochondrial biogenesis in skeletal muscle induced by different thermal environments.

## Introduction

Heat is a stress source of cells, and mitochondrial function is affected by heat. Skeletal muscle temperature is in the range of 35–36°C in normal conditions in humans, and high muscle temperature can be defined when temperatures exceed 40°C or even 42°C (Fiorenza et al., 2019). Mitochondria, as the most abundant organelles in skeletal muscle, are affected by temperature. Previous studies have found that high temperature can induce mitochondrial biogenesis in skeletal muscle, which is beneficial to mitochondrial oxidative phosphorylation (OXPHOS) in skeletal muscle (Liu and Brooks, 2012; Zoladz et al., 2016). Mitochondrial oxidation ability depends on sufficient mitochondria, so mitochondrial biogenesis is an important part for the mitochondrial function in skeletal muscle. Mitochondrial biogenesis requires mitochondrial DNA replication and transcription, as well as protein synthesis. Given that the OXPHOS system is located in the mitochondrial inner membrane, increased mitochondrial biogenesis adaptation in skeletal muscle ensures the enhancement of OXPHOS, which ultimately increases ATP production (Joseph et al., 2006).

Transient receptor potential vanilloid 1 (TRPV1) is a six transmembrane protein located on the sarcoplasmic reticulum (SR), which can be activated by high temperature (≥40°C), as well as capsaicin and acid poisoning (Nilius and Flockerzi, 2014). Another study also confirmed that 45°C activates TRPV1 in mouse skeletal muscle cells, which proves its sensitive response to high temperature (Lotteau et al., 2013). Activated TRPV1 releases Ca^2+^ into the cytoplasm, and increased intracellular Ca^2+^ regulates cascade signaling and upregulates mitochondrial biogenesis in tissues.

TRPV1 has been proved to exist in skeletal muscle (Obi et al., 2017). TRPV1 may be a key protein in response to mitochondrial biogenesis by high muscle temperature. The purpose of this review is to examine the roles of TRPV1 in high temperature-induced mitochondrial biogenesis and highlight the potential mechanism for understanding the relationship between high temperature and mitochondrial biogenesis in skeletal muscle.

## Overview on TRPV1

### Protein Structure of TRPV1

Transient receptor potential (TRP) cation channels are cellular sensors for a wide spectrum of physical and chemical stimuli, and mammalian species possess 28 TRP channels (Zheng, 2013). All TRP channels have six transmembrane segments (S1–S6), such as voltage-gated potassium channels; both the N and C termini are intracellularly located (Benemei et al., 2015). TRPV1 is one of the TRP cation channels that can be activated by heat (Zheng, 2013). The TPRV1 structure can be divided into three parts: the N- and C-termini in cells, and the six transmembrane segments with pore loop region formed between S5 and S6 (Juárez-Contreras et al., 2020). The N-terminus of TRPV1 contains calmodulin and ATP binding sites, which modulate the activation of the channel (Cao et al., 2013; Lee and Zheng, 2015). Sites on the N-terminus are capable of phosphorylation by protein kinases with S116 proposed to be the most critical phosphorylation site for PKA-dependent reduction of desensitization (Mohapatra and Nau, 2003). A linker domain connects the N-terminus to the transmembrane region via the pre-helical segment (pre-S1) and connects TPRV1 subunits together (Liao et al., 2013).

The C-terminus contains protein kinase C (PKC) phosphorylation sites and sites for binding calmodulin and phosphatidylinositol-4,5-bisphosphate (PIP2), which can modulate the activation of the C-terminus (Jara-Oseguera et al., 2008). In addition, the C-terminus region of TRPV1 as a physiologically important Ca^2+^- CaM-binding site is implicated in TRPV1 desensitization (Lau et al., 2012). Furthermore, the thermal sensing domain is localized within the C-terminus (Brauchi et al., 2007).

The transmembrane region of the TRPV1 subunit comprises six helical segments (S1–S6), in which S1–S4 contribute to the voltage-sensing domain, and S5–S6 contribute to the pore-forming domain (Liao et al., 2013). S1–S4 are connected to S5 and S6 through the connecting segment and act as a foundation, which allows the linker segment to move, contributing to pore opening and TRPV1 activation. The transmembrane region also contains binding sites for several ligands (Cao et al., 2013).

### TRPV1 Location in Skeletal Muscle

TPRV1 is highly permeable to Ca^2+^ by cloning the genes expressed in dorsal root ganglion and overexpressed in human embryonic kidney cells in 1997 (Caterina et al., 1997). TRPV1 is highly expressed in brain stem, midbrain, hypothalamus, and limbic system in central tissues and widely expressed in heart, fat, and skeletal muscle in peripheral tissues (Edwards, 2014). Myofibers from skeletal muscles are generally categorized into glycolytic type II (fast twitch muscle fiber) and oxidative type I (slow twitch muscle fiber); there are more TRPV1 in the oxidative type I than in the glycolytic type II (Luo et al., 2012). The intracellular localization of TRPV1 is different between tissues. For example, TRPV1 is present in both the SR and mitochondrial outer membrane in cardiomyocytes (Randhawa and Jaggi, 2015; Juárez-Contreras et al., 2020), whereas TRPV1 in skeletal muscle cells is localized on the SR (Xin et al., 2005). TRPV1 is enriched in longitudinal SR but not in the mixture of longitudinal SR and terminal cisternae, which is drawn from Western blot results from protein samples prepared by sucrose step gradient centrifuge (Lotteau et al., 2013). Moreover, immunofluorescence observation confirmed the localization of TRPV1 on the SR membrane rather than on the sarcolemma (Lotteau et al., 2013).

## TRPV1 Function in Skeletal Muscle

TRPV1 is susceptible to the cell environment temperature. High temperatures of 40–45°C can activate the TRPV1 channel (Venkatachalam and Montell, 2007). High muscle temperature can activate TRPV1 channels on the longitudinal SR. The Ca^2+^ leak from the SR to the cytoplasm might occur in two phases. In the first phase, activated TRPV1 releases Ca^2+^ from the SR to the cytoplasm (Vanden Abeele et al., 2019). In the second phase, elevated cytosolic Ca^2+^ triggers the activation of RyR1, which results in more prominent SR Ca^2+^ release (Lotteau et al., 2013).

TRPV1 channels are involved in the detection of cell environment temperature and play an important role in thermoregulation (Castillo et al., 2018). Studies have confirmed that the C-terminus of TRPV1 and the membrane proximal domain of its N-terminus are essential components of the temperature sensing machinery (Brauchi et al., 2006; Yao et al., 2011). Additionally, studies using pharmacological tools have revealed clear and highly reproducible effects of TRPV1 on thermoregulation (Garami et al., 2011). TRPV1 signals in the peripheral nerve system tonically suppress general locomotor activities, which may help bring down the body temperature (Garami et al., 2011). Therefore, TRPV1 channels are tonically active *in vivo* and regulate body temperature to prevent excessive elevation of body temperature.

### Sustained TRPV1 Activation Promotes Mitochondrial Biogenesis

Long-term activation of TRPV1 promotes mitochondrial biogenesis. Ca^2+^ is a general intracellular messenger and plays an important role in various physiological and biochemical reactions of cells (Chin, 2010). Maintaining intracellular Ca^2+^ homeostasis is essential for cell function. The expression of TRPV1 and cytosolic Ca^2+^ concentration in skeletal muscle cells increased in mice fed with capsaicin for 4 months, which increased the expression of peroxisome proliferator-activated receptor gamma coactivator-1α (PGC-1α), a master regulator in promoting mitochondrial biogenesis, and enhanced aerobic endurance capacity (Luo et al., 2012). The sustained activation of TRPV1 by injecting 10 μM capsaicin twice a day for 13 days promoted the release of SR Ca^2+^ and induced the mitochondrial biogenesis of skeletal muscle in mice (Ito et al., 2013).

**FIGURE 1:**
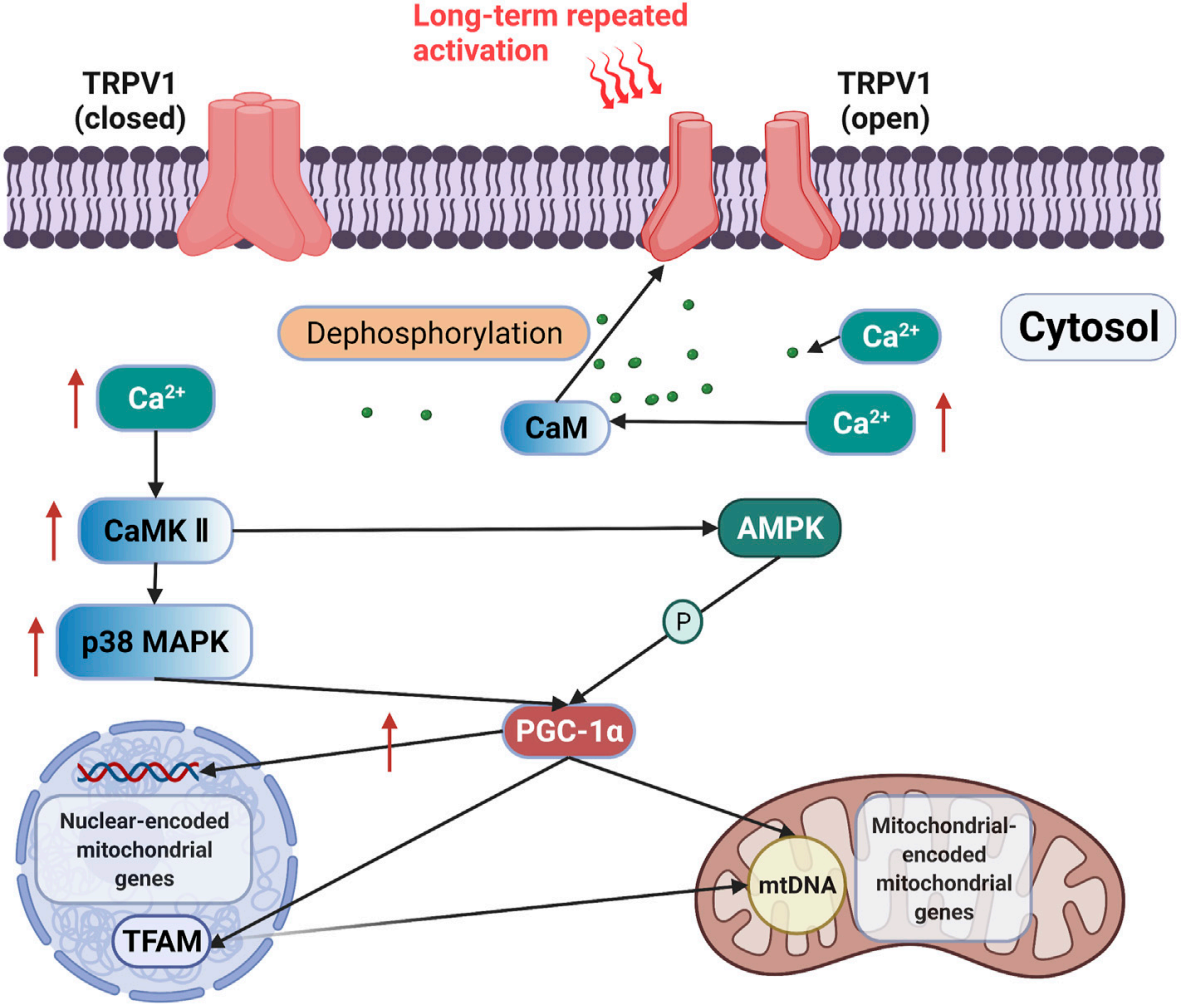
Signaling axis of TRPV1 is activated to improve mitochondrial biogenesis. Long-term activation of TRPV1 releases Ca^2+^ from the SR to the cytoplasm, and the increase in Ca^2+^ may induce PGC-1α expression through the activation of CaMKII. The activation of PGC-1α increases the expression of nuclear-encoded mitochondrial genes and upregulates mitochondrial DNA transcription and replication via TFAM (Islam et al., 2018).

Long-term activation of TRPV1 may promote mitochondrial biogenesis through the Ca^2+^-CaMKII-p38 MAPK-PGC-1α signaling axis (Figure 1). The sustained activation of TRPV1 by feeding mice with diet containing 0.01% capsaicin for 16 weeks upregulated PGC-1α expression and promoted mitochondrial biogenesis in kidney cells, which improved glomerular mitochondrial function (Wei et al., 2020). Luo et al. (Luo et al., 2012) subjected C57BL/6 J wild-type mice and TRPV1 knock-out mice to the dietary intervention of 0.01% capsaicin for 4 months. The results showed that sustained activation of TRPV1 by dietary capsaicin upregulated PGC-1α and increased mitochondrial content and ADP-stimulated respiratory functions of skeletal muscle in C57BL/6 J wild-type mice but not in TRPV1 knockout mice. Moreover, high TRPV1 expression in TRPV1-transgenic mice increased PGC-1α expression, oxidative fiber ratio, and exercise endurance. Therefore, chronic activation of TRPV1 in skeletal muscle can increase cytosolic Ca^2+^ concentration and PGC-1α contents, which can promote mitochondrial biogenesis and aerobic endurance capacity. In addition, studies have found that mitochondrial biogenesis is suppressed after TRPV1 inhibition. In septic mice, 30 mg/kg TRPA1 antagonist A-967079 inhibited the expression of TRPV1 in kidney cells; 1 week later, PGC-1α and mitochondrial transcription factor A (TFAM) protein levels were decreased by 68.3 and 53.15%, respectively, indicating that inhibition of TRPV1 reduced mitochondrial biogenesis signals (Zhu et al., 2018). PGC-1α in muscle is an essential regulator of mitochondrial biogenesis. High expression of PGC-1α in skeletal muscle of transgenic mice resulted in the increase of mitochondrial biogenesis and oxidative muscle fibers (Rowe et al., 2013), whereas the deletion of PGC-1α and PGC-1β in muscle led to a significant decrease in mitochondrial respiration, electron transport chain (ETC)/OXPHOS gene expression, and aerobic exercise performance (Terada et al., 2005). The above evidence showed that TRPV1 activation for a long period can promote mitochondrial biogenesis by upregulating PGC-1α expression in a Ca^2+^-dependent manner.

TRPV1-mediated Ca^2+^ release may induce PGC-1α expression through the activation of CaMKII. The activation of CaMKII enhances the phosphorylation level of p38 MAPK, which then initiates the transcription of PGC-1α, thereby promoting mitochondrial biogenesis (Akimoto et al., 2005). Kidney cells isolated from mice fed with 0.01% capsaicin for 16 weeks exhibited increased expression of TRPV1, CaMKII, and the phosphorylation levels of CaMKII (Wei et al., 2020). The activation effect of TRPV1 on 5′ AMP-activated protein kinase (AMPK) was abolished by knocking down CaMKII, but the inhibition of AMPK did not affect CaMKII phosphorylation by TRPV1 (Wei et al., 2020). These results indicated the presence of a TRPV1-Ca^2+^-CaMKII signaling axis. Therefore, chronic activation of TRPV1 may promote mitochondrial biogenesis through the Ca^2+^-CaMKII-p38 MAPK-PGC-1α signaling axis.

### Desensitization

Ca^2+^ concentration is one of the key regulators of TRPV1 desensitization because the persistent exposure of TRPV1 to Ca^2+^ stimulation can attenuate its responses (Zhao and Tsang, 2017). High intracellular Ca^2+^ concentration reduces TRPV1 activity through negative feedback and dynamically adjusts intracellular Ca^2+^ concentration (Shuba, 2020). Dephosphorylation by Ca^2+^-dependent phosphatase calcineurin is also involved in regulating TRPV1 desensitization (Bevan et al., 2014). Phosphorylation mediated by CaMKII, PKA, and PKC at several consensus sites has been reported to decrease Ca^2+^-mediated desensitization of TRPV1 (Vyklický et al., 2008). Therefore, TRPV1-mediated CaMKII activation may serve as positive feedback to amplify the signal. The biological significance of calmodulin-mediated reduction of TRPV1 activity and CaMKII-mediated reduction of TRPV1 desensitization is to regulate repeated stimulations of TRPV1 and maintain intracellular Ca^2+^ concentration.

## Conclusion

TRPV1 is sensitive to the temperature of the cell environment, and high temperatures of 40–45°C can activate the TRPV1 channel. Ca^2+^ is the key messenger regulating mitochondrial biogenesis in skeletal muscle by TRPV1. Long-term activation of TRPV1 may promote mitochondrial biogenesis through the Ca^2+^-CaMKII-p38 MAPK-PGC-1α signaling axis in skeletal muscle. Moreover, cellular Ca^2+^ concentration is one of the regulators of TRPV1 desensitization because the persistent exposure of TRPV1 to Ca^2+^ stimulation can attenuate its activity. CaMKII-, PKA-, and PKC-mediated phosphorylation at several consensus sites have been reported to decrease Ca^2+^-mediated desensitization of TRPV1.

## Future Perspectives

SR-located TRPV1 of skeletal muscle is functionally activated by heat in some local temperature elevation environments, such as muscle exercise or/and accompanied with muscle fatigue (Lotteau et al., 2013). A basal Ca^2+^ leak from the SR to the cytosol may occur through TRPV1 in these conditions.

The discovery of the TRPV1 channel highlights the potential effect of high temperature in improving mitochondrial biogenesis. The muscle temperature during long-term aerobic exercise always increases to 40°C, even to 42°C (Fiorenza et al., 2019), reaching the temperature activation threshold of TRPV1. Athletes working in heated environments, such as workshop, hot water immersion, and exercise environments, might receive some benefits in regard to skeletal muscle adaptation. Interestingly, the trapezius muscle from mice was subjected to 40°C heat stress for 20 min and isometric muscle contractions were elicited by electrical stimulation; heat stress-induced activation of TRPV1 was abolished by concomitant muscle contraction (Ikegami et al., 2019). However, comprehensive human studies on TRPV1 and heat-induced muscle mitochondrial biogenesis are still lacking. Effects of active heating and passive heating on the activation of TRPV1 by high muscle temperature also need to be further studied.
